# Suggestion of more suitable study designs and the corresponding reporting guidelines in articles published in the *Journal of Educational Evaluation for Health Professions* from 2021 to September 2022: a descriptive study

**DOI:** 10.3352/jeehp.2022.19.36

**Published:** 2022-12-26

**Authors:** Soo Young Kim

**Affiliations:** Department of Family Medicine, Kangdong Sacred Heart Hospital, Hallym University College of Medicine, Seoul, Korea; Hallym University, Korea

**Keywords:** Periodicals as topic, Policy, Research design, Research report

## Abstract

**Purpose:**

This study aimed to suggest a more suitable study design and the corresponding reporting guidelines in the papers published in the *Journal of Educational Evaluation for Health Professionals* from January 2021 to September 2022.

**Methods:**

Among 59 papers published in the *Journal of Educational Evaluation for Health Professionals* from January 2021 to September 2022, research articles, review articles, and brief reports were selected. The followings were analyzed: first, the percentage of articles describing the study design in the title, abstracts, or methods; second, the portion of articles describing reporting guidelines; third, the types of study design and corresponding reporting guidelines; and fourth, the suggestion of a more suitable study design based on the study design algorithm for medical literature on interventions, systematic reviews & other review types, and epidemiological studies overview.

**Results:**

Out of 45 articles, 44 described study designs (97.8%). Out of 44, 19 articles were suggested to be described with more suitable study designs, which mainly occurred in before-and-after studies, diagnostic research, and non-randomized trials. Of the 18 reporting guidelines mentioned, 8 (44.4%) were considered perfect. STROBE (Strengthening the Reporting of Observational Studies in Epidemiology) was used for descriptive studies, before-and-after studies, and randomized controlled trials; however, its use should be reconsidered.

**Conclusion:**

Some declarations of study design and reporting guidelines were suggested to be described with more suitable ones. Education and training on study design and reporting guidelines for researchers are needed, and reporting guideline policies for descriptive studies should also be implemented.

## Introduction

### Background/rationale

Reporting guidelines can be defined as “a checklist, flow diagram, or structured text to guide authors in reporting a specific type of research, developed using explicit methodology” [1]. Reporting guidelines have been developed for various study designs. A journal encourages authors to follow these guidelines because they help researchers explain their studies sufficiently to be evaluated by editors, reviewers, readers, and other researchers [2]. The *Journal of Educational Evaluation for Health Professionals* (JEEHP) is one of the academic journals that has recommended the use of reporting guidelines in writing manuscripts. Since 2021, JEEHP has adopted a journal policy that requires reporting guidelines to be described in the Methods section. Compliance with reporting guidelines is essential for enhancing transparency in journals. Therefore, the policies of journals related to reporting guidelines should not only have a simple declarative meaning but also mandate that authors accurately determine the study design of the manuscript, select appropriate reporting guidelines, and write the manuscript according to the reporting guidelines.

Studies related to reporting guidelines in academic journals have investigated the adoption rate of reporting guideline policies in academic journals [3] and the ratio of declaring reporting guidelines in the articles [4]. However, since these studies focused only on specific reporting guidelines such as CONSORT (Consolidated Standards of Reporting Trials), they do not provide a comprehensive overview of issues related to reporting guidelines. Especially not many academic journals mandate the declaration of reporting guidelines, so the case of JEEHP can be considered unique. In this regard, it would be meaningful to review whether the study design and the corresponding recommendation reporting guidelines have been adequately indicated in papers published in JEEHP.

### Objectives

The purpose of this study was to suggest the study design and the corresponding reporting guidelines in research articles, review articles, and brief reports published in JEEHP from January 2021 to September 2022. Specifically, the following were investigated: the proportiom of articles describing the study design; the proportion of articles describing reporting guidelines; the types of study design and corresponding reporting guidelines; and, a suggestion of a more suitable study design based on the study design algorithm for medical literature on interventions (DAMI), systematic reviews & other review types, and epidemiological studies overview.

## Methods

### Ethics statement

It is not a human study but a literature-based study. Therefore, neither approval by the institutional review board nor obtainment of informed consent is required.

### Setting

Articles in JEEHP published from January 2021 to September 2022 were searched for the presence of study design and corresponding reporting guidelines.

### Subjects

Research articles, review articles, and brief reports were included among the papers published during the period. Editorials, educational/faculty development materials, and technical reports were excluded.

### Variables

Study design and reporting guidelines were variables.

### Measurement

#### General characteristics of the included articles

The general characteristics assessed were as follows: (1) type of research (research article, review, brief report, case report); (2) description of study design: whether a description of the study design was present in the Methods section; and (3) type of study design: study design classified by the author (S.Y.K.) referring to research design classification systems, such as DAMI (a study design algorithm for medical literature on interventions) [5], systematic reviews & other review types (https://guides.temple.edu/c.php?g=78618&p=4156607), and the epidemiological studies overview (https://lo.unisa.edu.au/mod/book/view.php?id=646428).

#### Suggestion of a more suitable study design description

It was examined whether the study design classification described in the Methods section and the author’s (S.Y.K.) research classification were consistent.

#### Availability of reporting guidelines and amore suitable selection of reporting guidelines

It was examined whether the reporting guidelines indicated by the author were enough. The reporting guidelines were searched in EQUATOR (The EQUATOR Network and UK EQUATOR Centre, Oxford, UK) to determine whether they were available according to the most up-to-date classification of study designs. It was also examined whether the reporting guidelines indicated by the author were suitable.

Raw analysis data of 45 articles are available from Dataset 1.

### Bias

There was no selection bias.

### Study size

Sample size estimation is unnecessary since all target articles were included in the analysis.

### Statistical methods

Descriptive statistics were used.

## Results

### General characteristics of the included articles

Among the 59 papers published in JEEHP from 2021 to September 2022, the number of research articles, review articles, and brief reports was 45. Among the 45 publications, the most common type of publication was original articles (n=30, 66.7%), followed by reviews and brief reports. Out of 45 studies, 44 described the study design (97.8%), but only half indicated this information in the title or abstract. In the actual study design of the 45 papers re-classified by the expert (S.Y.K.), the most common study designs were descriptive cross-sectional surveys (n=14, 31.1%), followed by before-and-after studies (n=9, 20.0%), diagnostic accuracy tests (n=5, 11.1%), and non-randomized trials (n=4, 8.9%). Eighteen articles indicated the reporting guidelines in the Methods section (Table 1).

### Suggestion of a more suitable study design description

There were 25 articles (55.6%) in which the indicated study design matched a study design endowed by the expert (S.Y.K.). A descriptive cross-sectional survey was the most common study design that can be classified again. The authors of JEEHP described descriptive cross-sectional surveys using other study design terms (content analysis study, analysis study, comprehensive study, network analysis, mixed study; n=5, 25%). In other cases, the actual study design was a before-and-after study, but the authors described it as a comprehensive observational study, methodological study, or cross-sectional study (n=5, 25%). In addition, studies that could be classified as diagnostic studies were described as explicit studies, simulation studies, or other terms (n=4, 20%) (Table 2).

### Availability of reporting guidelines

Eighteen (40.0%) of the 45 articles indicated reporting guidelines (Table 1). The largest percentage was for diagnostic studies (STARD [Standards for the Reporting of Diagnostic Accuracy Studies]; n=5, 27.8%), followed by non-randomized trials (TREND [Transparent Reporting of Evaluations with Nonrandomized Designs]; n=4, 22.2%), systematic reviews (Preferred Reporting Items for Systematic Reviews and Meta-Analyses [PRISMA]; n=3, 16.7%), scoping reviews (PRISMA-ScR; n=2, 11.1%), and analytic observation studies (Strengthening the Reporting of Observational Studies in Epidemiology [STROBE]; n=2, 11.1%) in descending order (Table 3).

However, 27 of the 45 papers (60%) did not indicate corresponding reporting guidelines. The largest percentage of these were descriptive cross-sectional surveys (n=14, 51.9%), followed by before-and-after studies (n=9, 33.3%), and narrative reviews (n=3, 11.1%) (Table 4).

### Suggestion of reclassification of reporting guideline

Of the 18 reporting guidelines indicated by authors, 8 (44.4%) were well-matched. Among the 10 articles in which a re-selection of reporting guidelines was suggested, the most frequent was the application of STROBE to descriptive studies (n=5, 50%). In addition, there were cases where STROBE was applied to before-and-after studies (n=4, 40.0%) and a randomized controlled trial (n=1, 10.0%) (Table 5).

## Discussion

### Key results

Out of 45 research articles in JEEHP from January 2021 to 2022, 44 described the study designs (97.8%), but only 25 (55.6%) were considered well-matched study designs. Other 19 study design descriptions that could be re-classified mainly occurred in studies in which the actual study designs were before-and-after studies, diagnostic research, and non-randomized trials. Of the 18 reporting guidelines indicated by authors, 8 (44.4%) were well-matched. Other descriptions of reporting guidelines that can be reselected included the application of STROBE to non-applicable research (descriptive studies, before-and-after studies, and randomized controlled trials).

### Interpretation

Out of 44 study design descriptions, 41.3% recorded in the Methods sections of papers published in JEEHP needs to be, and 60.0% of the described reporting guidelines also need to be re-classified. If a study design does not match the content, the reporting guidelines will not match. Therefore, these 2 results are correlated.

### Comparison with previous studies

No previous reports examined the match of study designs with content and reporting guidelines simultaneously. However, one previous study examined the appropriateness of the reporting guidelines described in papers. Innocenti et al. [4] stated that reporting guidelines were in 17.5% of rehabilitation research articles, and among these studies, 48.6% (95% confidence interval, 32%–65.1%) used study designs appropriately. In the present study, 18 (40.0%) out of 45 studies declared the use of reporting guidelines, which is a higher rate than in previous studies, but the proportion of well-matched use was 44.4% out of 18 reporting guideline descriptions. No previous paper has suggested reasons why studies did not declare reporting guidelines. According to the results of this paper, the problem is that reporting guidelines sometimes cannot be declared because many study designs do not have corresponding reporting guidelines. Only 40.0% of the studies analyzed herein could select the reporting guidelines. Therefore, if reporting guidelines are developed for descriptive cross-sectional surveys or before-and-after studies, the rate of declaration of reporting guidelines may increase. The study design description in 20 out of 45 articles needs to be re-classified in the present study. Previous studies have examined whether the study design described in the Methods section was appropriate. One study investigated the appropriateness of the classification of research designs published in 3 dermatology journals and found that 26.5% of observational studies had inappropriately described research designs [6]. In 4 Asian dermatology journals in Science Citation Index-Expanded, 72 publications (40.7%) of the 177 papers in 2018 declared their research design. Of those, 23 articles (32.0%) demonstrated differences between the author-reported and the actual study designs [7]. The results of this study can be seen as similar to or slightly higher than those of previous studies.

### Limitations/generalizability/suggestions

There were limitations to this study. First, judgments of the match of study design with content and the corresponding reporting guidelines involved some subjectivity, although the author has expertise in classifying study designs. Second, the small number of target papers is another limitation. The analysis was conducted only on papers published after 2021, and a more general conclusion would have been possible if the period had been extended.

### Conclusion

According to the above result, education and training on both study design and reporting guidelines are necessary. In addition, it may be necessary to establish a more realistic reporting guideline policy that reflects the actual development of reporting guidelines. The proportion of papers published in JEEHP after 2021 that declared reporting guidelines was not high, and the match of the description of the study design with content and reporting guidelines was also not high. Education and training on study design and reporting guidelines for researchers are needed. Reporting guideline policies should also be implemented considering the current development status of reporting guidelines.

## Figures and Tables

**Table 1.** General characteristics of the included articles (n=45)

Characteristic	No. (%)
Publication year	
2021	26 (57.7)
2022	19 (41.3)
Type of article	
Research	30 (66.7)
Review	8 (17.8)
Brief report	6 (13.3)
Case report	1 (2.2)
Description of study design	
Yes	44 (97.8)
No	1 (2.2)
Description of study design at	
Title	25 (55.6)
Abstract	24 (53.3)
Methods	37 (82.2)
Study design	
Descriptive cross-sectional survey	14 (31.1)
Before-and-after study	9 (20.0)
Diagnostic accuracy test	5 (11.1)
Non-randomized trial	4 (8.9)
Systematic review	3 (6.7)
Narrative review	3 (6.7)
Scoping review	2 (4.4)
Qualitative methodology	1 (2.2)
Comparative before-and-after study	1 (2.2)
Historical cohort study	1 (2.2)
Randomized controlled trial	1 (2.2)
Technical report	1 (2.2)
Description of the reporting guideline	
Yes	18 (40.0)
No	27 (60.0)

**Table 2.** The suggestion of a more suitable description of study design

	No. (%)
Well-matched description of the study design	
Yes	25 (55.6)
No	20 (44.4)
Study design description that needs to be classified again (n=20)	
Suggested description of a descriptive cross-sectional survey	5 (25.0)
Suggested description of a before-and-after study	5 (25.0)
Suggested description of a diagnostic accuracy study	4 (20.0)
Suggested description of a non-randomized trial	3 (15.0)
No study design description	1 (5.0)
Other	2 (10.0)

**Table 3.** Study designs with selectable reporting guidelines (n=18)

Study design	Reporting guidelines	No. (%)
Diagnostic study	STARD	5 (27.8)
Non-randomized trial	TREND	4 (22.2)
Systematic review	PRISMA	3 (16.7)
Scoping review	PRISMA-ScR	2 (11.1)
Analytic observational study	STROBE	2 (11.1)
Randomized controlled trial	CONSORT	1 (5.6)
Qualitative study	COREQ	1 (5.6)
Total		18 (100.0)

STARD, Standards for the Reporting of Diagnostic Accuracy Studies; TREND, Transparent Reporting of Evaluations with Nonrandomized Designs; PRISMA, Preferred Reporting Items for Systematic Reviews and Meta-Analyses; PRISMA-ScR, PRISMA-scoping reviews; STROBE, Strengthening the Reporting of Observational Studies in Epidemiology; CONSORT, Consolidated Standards of Reporting Trials; COREQ, Consolidated Criteria for Reporting Qualitative Research.

**Table 4.** Study designs with no corresponding reporting guidelines (n=27)

Study design	No. (%)
Descriptive cross-sectional survey	14 (51.9)
Before-and-after study	9 (33.3)
Narrative review	3 (11.1)
Technical report	1 (3.7)
Total	27 (100.0)

**Table 5.** Reporting guideline selection to be re-classified (n=18)

	No. (%)
Well-matched reporting guideline	
Yes	8 (44.4)
No	10 (55.6)
Reselection of a reporting guideline suggested (n=10)	
STROBE application to a descriptive study	5 (50.0)
STROBE application to a before and after study	4 (40.0)
STROBE application to a randomized controlled trial	1 (10.0)

STROBE, Strengthening the Reporting of Observational Studies in Epidemiology.

## Footnotes

**Data availability**
Data files are available from Harvard Dataverse: https://doi.org/10.7910/DVN/TYWWZ2 Dataset 1. Data file containing the study design and guidelines of articles published from 2021 to September 2022 in the *Journal of Educational Evaluation for Health Professions*.

## Supplementary materials

Supplementary files are available from Harvard Dataverse: https://doi.org/10.7910/DVN/TYWWZ2

Supplement 1. Audio recording of the abstract.
